# No Significant Reduction of Circulating Endothelial-Derived and Platelet-Derived Microparticles in Patients with Psoriasis Successfully Treated with Anti-IL12/23

**DOI:** 10.1155/2016/3242143

**Published:** 2016-04-10

**Authors:** Ji-Chen Ho, Chih-Hung Lee, Shang-Hung Lin

**Affiliations:** Department of Dermatology, Kaohsiung Chang Gung Memorial Hospital and Chang Gung University College of Medicine, Ta-Pei Road 123, Niao-Sung, Kaohsiung 83301, Taiwan

## Abstract

Psoriasis is associated with atherosclerosis, in which circulating microparticles play an important role. In severe psoriasis, there was an increase of endothelial- and platelet- microparticles which could be decreased by anti-TNF*α*. However, whether anti-IL-12/23 treatment would decrease the level of microparticles remains unknown. Our study showed that, despite the clinical improvement of psoriasis after IL-12/13 blockage, the increased levels of circulating CD41a and CD31 microparticles were unchanged after anti-IL-12/23. This result suggested that anti-IL12/23 treatment may not alter the development of cardiovascular disease in patients with psoriasis.

## 1. Introduction 

Psoriasis is a common and chronic inflammatory disease. It affects not only skin but also several organ systems, including musculoskeletal system, microcirculations, and metabolic system. Patients with psoriasis have a high risk to develop atherosclerosis, leading to cardiovascular events and stroke [1, 2]. The patients with severe psoriasis have a shortened lifespan [3]. The detailed pathophysiological mechanisms by which psoriatic patients develop atherosclerosis remain unclear; however, the endothelial dysfunction, uncontrolled inflammation, and persistent thrombosis might be involved.

Microparticles (MPs) are membrane vesicles of 0.1 to 1 um in diameter generated from activated or apoptotic cells. The contents of MPs from leukocytes contain IL-1, CD40L, and ICAM1, all of which are associated with the vascular inflammation and development of unstable atherosclerotic plaques. Besides atherosclerosis [4], MPs are elevated in several chronic inflammatory diseases because of the process of apoptosis, such as systemic lupus erythematous and rheumatoid arthritis [5, 6]. The inflammatory nature of atherosclerosis suggests that chronic inflammation in psoriasis may accelerate atherosclerosis through repetitive vascular injury.

It has been reported that endothelial cell- and platelet-derived MPs are increased in peripheral blood from patients with psoriasis [7–9]. In fact, the presence of increased endothelial cell and platelet activation with cell turnover reflecting increased MP levels may contribute to the heightened atherogenesis associated with psoriasis [10]. Imbalanced endothelial injury and repair, reflecting increase in endothelial MPs in patients with psoriasis, are associated with preclinical atherosclerosis [11]. In addition, Pelletier et al. reported that MPs are significantly decreased in psoriatic patients successfully treated with TNF-*α* inhibitors (etanercept, adalimumab, and infliximab) [12]. A new effective biologic targeting IL12/23 axis, ustekinumab, was developed for the treatment for psoriasis. It is quite effective in treating both psoriasis and psoriatic arthritis. According to current evidence, the benefits and risks of cardiovascular disease in psoriatic patients with ustekinumab treatment are inconclusive [13, 14]. Whether this agent decreased endothelial MPs in patients with psoriasis was unknown. The aim of our study is to evaluate whether effective anti-IL12/23 treatments would reduce endothelial cells and platelets MPs.

## 2. Methods 

### 2.1. Inclusion of Patients with Psoriasis and Healthy Controls

Eleven patients (age ranges 38 to 68 years, male : female = 9 : 2) with severe psoriasis who received anti-IL12/23 (ustekinumab, UST) treatments and healthy controls between August 2014 and July 2015 were included. These patients received UST 45 mg at 0 and 1 month. UST is humanized monoclonal anti-p40 antibody (subunits of IL12 and IL23). The psoriatic patients who had pregnancy or infection (such as tuberculosis or sepsis) were excluded. There were nine healthy controls (age ranges 34 to 59 years, male : female = 7 : 2). The patients who were diagnosed as psoriasis by two dermatologists were included in the psoriatic group. Patients in the control group were examined thoroughly to make sure no psoriatic lesions were found by two dermatologists. Age, sex, lipid profiles, and blood pressure were recorded for all subjects. Psoriatic patients were further recorded for the past history of diabetes, hypertension, psoriatic arthritis, myocardial infarction, and cerebrovascular disease after the onset of psoriasis. The previous treatments were also recorded. Psoriasis severity was measured by PASI (psoriasis area severity index). This study was approved by the Institutional Ethics Committee of Chang Gung Memorial Hospital and conducted following the ethical guidelines of the Declaration of Helsinki.

### 2.2. Measurement of Microparticles

The blood levels of MPs were evaluated. In patients with psoriasis, we measured MPs in the baseline and 3 months after second UST injection. For control group, we measured MPs in the baseline. We compared the MPs levels among normal control, psoriasis without comorbidities, and psoriasis with at least two comorbidities (including cerebrovascular disease, diabetes mellitus, hypertension, hyperlipidemia, and psoriatic arthritis). 1.5 mL of blood was obtained and collected in citrated tubes after discarding the first 1 mL of blood. The platelet-poor plasma (PPP) was then collected, aliquoted, and stored at −80°C until further analysis. Reagents for MPs, including FITC-Annexin V (Catalog number 640906), PE-Cyanine7-CD31 (Catalog number 555446, clone WM59), and APC-CD41a (Catalog number 559777, clone HIP8), were obtained from eBioscience. Aliquots of 100 *μ*L of each sample were stained and added to bead-containing TruCount tubes (Catalog 340334, BD). Annexin Buffer (10 mmol/L Hepes, pH 7.4, 140 mmol/L NaCl, and 2.5 mmol/L CaCl2), double-filtered by a 0.22 lm filter, was added to each tube to make the total volume 250 *μ*L. These samples were performed on a BD Accuri C6 Cytometry (BD Biosciences, San Jose, CA).

For the detection of microparticles by flow cytometry, an initial microparticle-size gate was set with the help of 1.0 *μ*m and 3.0 *μ*m calibration particles (Spherotech, Chicago). Then we defined microparticles as particles that were less than 1.0 *μ*m in diameter. The microparticles that had positive staining for both Annexin V and surface markers (CD31 or CD41a) separate true events from background noise and unspecific binding of antibodies to debris. Isotype controls were used as negative control in this experiment. The absolute count using TruCount tubes was calculated from the appropriate dot plot values entered into a spreadsheet that was formatted to use the formula [(no. of events in quadrant containing cell population)/(no. of events in absolute count bead region)] × [(total no. of absolute count beads [49200])/(test volume [100 *μ*L])].

### 2.3. Statistical Analysis

Age and the levels of CD41a and CD31 MPs between psoriatic patients and normal control were compared by *t*-test. The changes of CD41a and CD31 positive MPs at the baseline and 4 months after treatments were analyzed by paired *t*-test. A *p* value less than 0.05 (2 tails) was considered to be of statistical significance.

## 3. Results

### 3.1. Dermographic Data

11 patients with severe psoriasis and 9 normal controls were recruited (Table 1).

The psoriatic patient group included 9 males and 2 females with an average age of 49 years. All the psoriatic patients receiving anti-IL12/23 failed at least two of three systemic drugs (acitretin, methotrexate, and cyclosporine) for 3 months and failed narrow band UVB phototherapy for 3 months. The average PASI score before anti-IL12/23 was 22. The average disease duration was 13 years. Three psoriatic patients had psoriatic arthritis, 5 psoriatic patients had hypertension, 2 psoriatic patients had diabetes, 8 psoriatic patients had hyperlipidemia, and 1 psoriatic patient had cerebrovascular disease. The normal controls included 7 males and 2 females with an average age of 48 years. In patients with psoriasis, after UST 45 mg at 0 and 1 month, the PASI severity score improved significantly (from 22.2 to 6.3, *p* < 0.05) (Table 1).

### 3.2. Higher CD41a and CD31 Positive MPs in Patients with Psoriasis Compared to Normal Control

**Figure 1:**
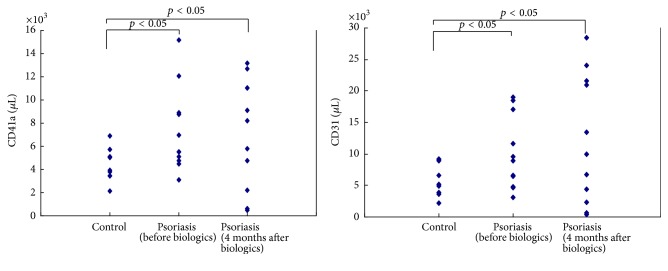
Increased levels of circulating CD41a and CD31 MPs in patients with psoriasis.

**Figure 2:**
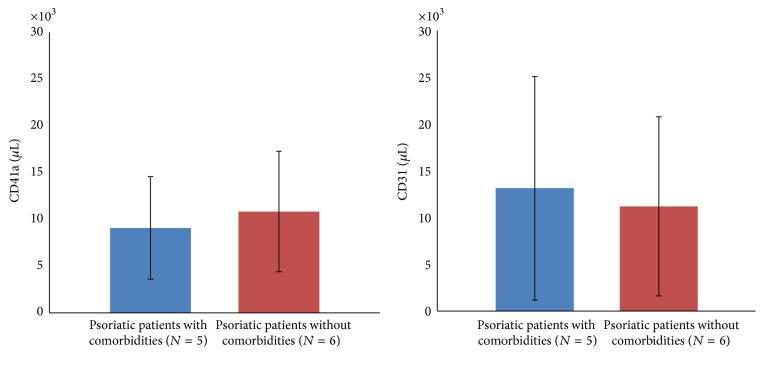
The levels of CD41a and CD31 MPs did not differ among psoriatic patients with comorbidities and without comorbidities.

Consistent with previous studies, we found that there were higher concentrations of CD41a (7305/*μ*L versus 4398/*μ*L, *p* < 0.05) and CD31 (4978/*μ*L versus 10036/*μ*L, *p* < 0.05) positive MPs corresponding to platelet and endothelial cell in psoriasis patients as compared with controls (Figure 1). Considering the role of MPs in the endothelial injury, we compared the levels of CD41a and CD31 MPs in psoriatic patients with or without comorbidities related to atherosclerosis. It turned out that the levels of CD41a and CD31 MPs were not different significantly in psoriatic patients with or without comorbidities (CD41a: 9080/*μ*L versus 10833, *p* > 0.05; CD31: 7798/*μ*L versus 6893/*μ*L, resp., *p* > 0.05) (Figure 2).

### 3.3. No Significant Reduction of the Levels of MPs among Patients with Psoriasis Successfully Treated with Anti-IL12/23 Treatment

**Figure 3:**
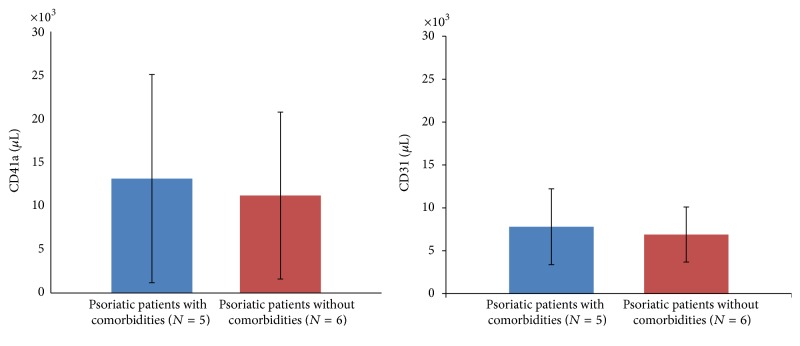
IL12/23 blockage did not decrease the level of circulating CD41a and CD31 MPs in patients with severe psoriasis despite clinical improvement.

Despite the effective reduction in PASI score and improvement in disease severity, the concentrations of CD41a (7305/*μ*L versus 7198/*μ*L, *p* > 0.05) and CD31 (10036/*μ*L versus 12075/*μ*L, *p* > 0.05) positive MPs were not different significantly in patients with psoriasis before and 4 months after UST (Figure 1). The levels of CD41a and CD31 MPs were not different significantly in psoriatic patients with comorbidities and without comorbidities 4 months after UST (CD41a: 13131/*μ*L versus 11195/*μ*L, *p* > 0.05; CD31: 7611/*μ*L versus 6854/*μ*L, resp., *p* > 0.05) (Figure 3).

## 4. Discussion

This is the first study to investigate the levels of MPs in patients with severe psoriasis after successful UST treatment. Our results showed that the levels of CD41a and CD31 MPs were consistently higher in patients with severe psoriasis as compared to those in normal controls. The levels of CD41a and CD31 MPs were not different significantly in psoriatic patients with or without comorbidities. Despite the improvement of clinical PASI score after UST treatment, the levels of CD41a and CD31 MPs did not decline significantly.

As a systemic inflammatory disease, psoriasis is associated with metabolic syndrome, including diabetes, obesity, hyperlipidemia, atherosclerosis, cardiovascular disease, and stroke [15]. These diseases of metabolic syndrome are associated with endothelial dysfunction, inflammation, and thrombosis. Microparticles, as membrane vesicles in the circulation derived from activated and apoptotic cells, are associated with many risk factors for vascular diseases [16]. MPs induce noxious responses and endothelial dysfunction by activation of procoagulant and proinflammatory pathways [17]. The platelet (CD41a) MPs could also inflict endothelial cell damage via induction of inflammation and impairment of endothelial-dependent vasodilation [18]. In acute myocardial infarction, platelet-derived MPs bind to the endothelium and facilitate thrombus propagation [19]. Similarly, CD 31+ endothelial MPs contribute to vascular homeostasis [20]. In fact, CD31 functions as an adhesion molecule receptor that facilitates leukocyte trafficking across the endothelial layer [21].

This study showed that CD31 and CD41a microparticles in patients with severe psoriasis did not decrease despite improved PASI after UST treatment. One report showed the decreased platelet and endothelial MPs with clinical improvement after 3-month anti-TNF-*α* agents in patients with psoriasis. According to current clinical studies, anti-TNF-*α* agents offer better evidence of benefit to lower the risk of cardiovascular diseases in patients with psoriasis. Further long-term study is necessary to quantify the benefits of cardiovascular diseases associated with UST treatment [14]. An in vitro study incubating human leukocytes and endothelial cells in a flow chamber showed that anti-TNF-*α* drugs inhibit the leukocyte recruitment induced by a wide variety of stimuli that act on both the endothelium and leukocytes. On the other hand, UST did not affect leukocyte recruitment induced by any of the endothelial stimuli. These findings suggest that anti-TNF-*α* drugs have the capacity to influence cardiovascular risk by ameliorating vascular inflammation [22]. The increased endothelial MPs levels could result from TNF-*α* in favor of endothelial MPs generation [23]. From these studies, the anti-TNF-*α* may be effective to reduce cardiovascular risk through the reduction of circulating microparticles. However, the role of ustekinumab in the MP production warrants a long-term follow-up.

This study had several limitations. First, the case number is small. Second, the follow-up duration is only 4 months, although PASI score has been improved significantly. A longer follow-up period may be required to observe the subtle changes of MPs after UST treatment.

## 5. Conclusions

The study concluded that the levels of CD41a and CD31 MPs were consistently higher in patients with severe psoriasis as compared to those in normal controls. The levels of CD41a and CD31 MPs were not different significantly in psoriatic patients with or without comorbidities. Despite the improvement of clinical PASI score after UST treatment, the levels of CD41a and CD31 MPs did not decline in parallel significantly.

## Figures and Tables

**Table 1:** Demographics of patients with psoriasis and normal controls.

	Age	Sex	PSA	HTN	DM	CAD	CVD	Dyslipidemia	PASI (initial)	PASI (4 months)	Disease duration	Previous medications	Phototherapy
PS01	40	M	−	+	+	−	−	+	36.7	28	5	MTX, cyclosporine	+
PS02	53	M	−	+	−	−	−	−	13	1.8	17	Acitretin, cyclosporine	+
PS03	58	M	−	+	+	−	+	+	17.4	3.6	16	Acitretin, MTX, cyclosporine	+
PS04	38	F	+	−	−	−	−	+	34.7	1.2	9	Acitretin, MTX	+
PS05	56	M	+	+	−	−	−	−	16.1	5.4	8	Acitretin, MTX	+
PS06	41	M	−	−	−	−	−	+	15.2	12.3	9	Acitretin, MTX, cyclosporine	+
PS07	49	M	−	−	−	−	−	+	18.6	7	12	Acitretin, MTX	+
PS08	68	F	+	+	−	−	−	+	23.8	2.1	20	Acitretin, MTX, cyclosporine	+
PS09	48	M	−	−	−	−	−	−	13.2	0.1	19	Acitretin, cyclosporine	+
PS10	38	M	−	−	−	−	−	+	13	2	8	Acitretin, MTX	+
PS11	51	M	−	−	−	−	−	+	42.2	5.6	20	Acitretin, MTX	+

Average	49								22.2	6.3^*∗*^	13		

C01	59	M	−	−	−	−	−	−					
C02	42	M	−	−	−	−	−	−					
C03	56	M	−	−	−	−	−	−					
C04	34	M	−	−	−	−	−	−					
C05	45	F	−	−	−	−	−	−					
C06	49	F	−	−	−	−	−	−					
C07	39	M	−	−	−	−	−	−					
C08	55	M	−	−	−	−	−	−					
C09	54	M	−	−	−	−	−	−					

Average	48												

PS: psoriasis; C: control; PSA: psoriatic arthritis; DM: diabetes; CAD: cardiovascular disease; CVD: cerebral vascular disease; PASI: psoriatic activity severity score; MTX: methotrexate. ^*∗*^ *p* < 0.05.
